# Implementation and Validation of Titratable Cysteine in GROMACS-Based Constant-pH Molecular Dynamics

**DOI:** 10.1021/acs.jctc.6c00640

**Published:** 2026-06-08

**Authors:** Riccardo Capelli

**Affiliations:** Department of Biosciences, 9304 Università degli Studi di Milano, Via Celoria 26, I-20133 Milan, Italy

## Abstract

Cysteine is a chemically versatile amino acid whose protonation state is central to catalysis, redox regulation, and covalent ligand recognition. Despite the growing availability of constant-pH molecular dynamics (CpHMD) methods, cysteine is not included among the standard titratable residues of the GROMACS-based λ-dynamics implementation developed by Aho, Buslaev, and co-workers. Here, we introduce a titratable cysteine residue, CYST, for this framework and integrate it into the phbuilder workflow. The implementation describes the thiol/thiolate equilibrium of reduced cysteines within a fixed redox topology, while disulfide-linked cysteines are retained as nontitratable covalent states defined *a priori*. The new residue was calibrated on an ALA-CYS-ALA (ACA) tripeptide by refining the correction potential required to remove the intrinsic force-field bias along the λ coordinate. The resulting model reproduces the expected sigmoidal titration behavior of the peptide and yields a *pK*_*a*_ of 8.33, matching the target value adopted in the calibration procedure. Transferability to proteins was then assessed using two complementary benchmarks. First, six engineered single-cysteine mutants of acyl-coenzyme A binding protein (ACBP) were used as a set of noncatalytic cysteines spanning different local environments. The calculations reproduce the overall high-*pK*_*a*_ regime of these residues, while also showing that quantitative accuracy depends sensitively on the local conformational ensemble sampled around the introduced cysteine. Second, the implementation was applied to the reactive Cys106 of DJ-1, placing its titration in the correct acidic regime. Overall, this work establishes a practical cysteine extension of the GROMACS CpHMD framework for biomolecular simulations.

## Graphical abstract

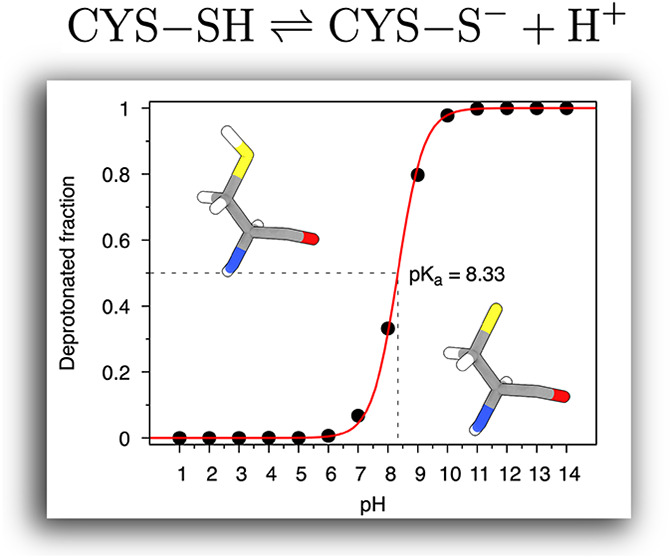

## Introduction

Cysteine is one of the most chemically versatile amino acids in proteins. Owing to its thiol side chain, it can participate in disulfide-bond formation, metal coordination, redox processes, and nucleophilic catalytic steps.^1–3^ In many of these contexts, the relevant reactive species is the deprotonated thiolate, whose population is determined by the cysteine *pK*_*a*_ and by the local electrostatic and hydrogen-bonding environment.^4,5^ Accurate modeling of cysteine protonation is therefore important both for understanding enzymatic and regulatory mechanisms and for predicting reactive cysteines in covalent drug discovery.^5,6^ At the same time, cysteine presents a unique challenge because, besides thiol/thiolate protonation equilibria, it can also form disulfide bridges, corresponding to a different covalent topology.^1,2^

Given the dynamic nature of cysteine protonation, the limitations of classical fixed-protonation representations of this amino acid are evident. A central development underlying constant-pH simulations was the introduction of λ-dynamics,^7^ in which an alchemical coordinate is treated as a dynamical variable and propagated together with the atomic degrees of freedom. This idea was later adapted to constant-pH molecular dynamics through continuous titration coordinates,^8^ and subsequently extended to include proton tautomerism in titratable residues such as histidine.^9^ The GROMACS implementation of Aho, Buslaev and co-workers^10,11^ belongs to this continuous λ-dynamics family of CpHMD methods, resulting particularly attractive because the computational cost of the constant-pH treatment does not depend on the number of titratable sites in the system. In addition, the same framework is accompanied by phbuilder,^12^ a workflow for automated setup of CpHMD simulations and for the introduction of new titratable residues or molecules. This implementation has already been successfully applied to ion channels,^13,14^ G-protein-coupled receptors,^15,16^ and pH-dependent activity in enzymes.^17^

Despite the versatility of this approach, the residues currently available out of the box are Asp and Glu, Lys and Arg, and histidine with its three physiologically relevant protonation states, while cysteine is not included.

Here, we parametrized cysteine in the modified CHARMM36 force field introduced in the original GROMACS CpHMD implementation.^11^ We show that this parametrization accurately describes the titration behavior of an ALA-CYS-ALA tripeptide (ACA). Next, we evaluate the predicted *pK*_*a*_ values of six single-cysteine mutants of bovine acyl-coenzyme A binding protein (ACBP), chosen as a benchmark set of noncatalytic cysteines. Lastly, we target Parkinson disease protein 7 (DJ-1), which contains a reactive oxidation-sensitive cysteine with a known *pK*_*a*_ shift.

In the present implementation, we restrict the constant-pH treatment to reduced cysteines and retain disulfide bridges as fixed covalent bonds defined *a priori* from the prepared input structure. The presented model therefore targets protonation equilibria within a fixed redox topology and does not attempt to describe disulfide formation or cleavage during the simulation.

## Methods

### CpHMD and MD Simulations

For all systems considered in this work, we employed GROMACS[Bibr ref18] 2024.4 for standard MD simulations and the CpHMD-modified GROMACS 2021 version^10,11^ for CpHMD simulations. Proteins were modeled with the CpHMD extension of the CHARMM36 force field (making all the Arg, Lys, His, Glu, Asp, and Cys in their titrable form for CpHMD runs), and water with the CHARMM36-compatible TIP3P model. Temperature and pressure were maintained using the velocity-rescale thermostat^19^ (coupling time 0.5 ps) and the cell-rescale barostat^20^ (isotropic coupling, coupling time 5 ps, compressibility 4.5 × 10^–5^ bar^–1^, reference pressure 1 bar), respectively. Bond lengths involving hydrogen atoms were constrained with the LINCS algorithm.^21^ Short-range electrostatic interactions were treated with the particle mesh Ewald method,^22^ using a real-space cutoff of 1.2 nm and a Fourier grid spacing of 0.14 nm. van der Waals interactions were truncated at 1.2 nm, with a force-switch modifier applied from 1.0 to 1.2 nm.

### ACA Tripeptide Setup and Cysteine Calibration

We generated the ACA tripeptide using tleap from AmberTools25.^23^ ACA was then placed in a cubic box with side length 5.17 nm, corresponding to a minimum solute-box distance of 1.8 nm, and solvated with 4500 water molecules and two buffer particles which have a dynamic charge from −0.5 to 0.5 e, allowing to maintain box neutrality in case of protonation state change. The system was prepared for CpHMD simulations using phbuilder.^12^

In the present CYST model, following the original implementation of CpHMD,^10^ the Lennard-Jones parameters are not interpolated along the λ coordinate. The residue is based on the protonated thiol topology, and the CpHMD transformation acts only on the partial charges of the atoms included in the titratable group. Thus, the van der Waals parameters remain those assigned by the CHARMM36 cysteine topology, whereas the charges of CB, SG, and HG1 are interpolated between the thiol and thiolate states. This choice follows the fixed-topology parametrization strategy used in the GROMACS CpHMD/phbuilder framework.

After steepest-descent energy minimization (tolerance 1000 kJ mol^–1^ nm^–1^), restrained NVT (1 ns) and NPT (10 ns) simulations were carried out with position restraints on all heavy atoms and cysteine in its thiol state.

Calibration of the dvdl_1 correction potential for CYST was performed on ACA following the phbuilder parametrization workflow. Briefly, the system was simulated in 13 fixed-λ windows from −0.1 to 1.1 (step 0.1), and the resulting ⟨*dV*/*dλ*⟩_λ_ values were fitted with a fifth-degree polynomial to obtain an initial estimate of the correction potential. The intrinsic reference *pK*_*a*_ for cysteine was set to 8.33, following the free-cysteine/reference-table *pK*_*a*_ scale used for the present CpHMD parametrization. We note that higher values, such as 8.55 ± 0.03, have been reported for blocked alanine peptide models of cysteine,^24^ and therefore reflect a different reference-state convention rather than an inconsistency in the parametrization. The resulting parametrization was then validated through ten independent 100 ns simulations at *pH* = *pK*_*a*_ = 8.33.

After calibration of the correction potential, we computed the full ACA titration curve by running a constant-pH simulation ladder from pH 1 to 14 in steps of 1 pH unit, covering the full titration range of the model cysteine. Each pH point was simulated at 300 K in triplicate, with 100 ns per replica, for a total sampling time of 4.2 μs.

### Analysis of Titration Curves

The deprotonated fraction at each pH value was computed from the corresponding λ trajectories. For each replica, frames with λ < 0.2 were assigned to the protonated state and frames with λ > 0.8 to the deprotonated state, while intermediate values were excluded from the state counting. The deprotonated fraction at each pH was then obtained by pooling the end point populations over the replicas, removing the first 50% of the trajectory to minimize the nonequilibrium initial part of the trajectory. The resulting titration curve was then fitted to the Henderson–Hasselbalch equation and a bootstrap approach was implemented to estimate confidence intervals (see Supporting Information).

### Protein Systems

#### ACBP Mutants

Starting coordinates were taken from the NMR structure of bovine acyl-coenzyme A binding protein (PDB 1NTI,^25^ model 1 of 20). Single-cysteine mutants were generated by replacing the corresponding target residue with cysteine. All ACBP simulations were performed at 298 K and 50 mM NaCl, consistently with the experimental conditions used for the NMR titration measurements.^26^ To maintain charge neutrality, 40 buffer particles have been added in the simulation box.

After steepest-descent energy minimization (tolerance 1000 kJ mol^–1^ nm^–1^), restrained NVT (1 ns) and NPT (10 ns) simulations were carried out, keeping the λ values fixed at their expected value at pH 7. For the nonrelaxed set of simulations, we performed the CpHMD preparation with phbuilder to the mutated systems and run a pH ladder from pH 6 to 12 in steps of 1 pH unit, all in triplicate for 100 ns per replica. To relax the initial conformation we performed a conventional MD run for 500 ns of the system with the protonation states chosen to represent pH 7 conditions. We underline that this step has to be considered as a structural prerelaxation step before CpHMD, rather than as part of the protonation-state sampling protocol. This step was intended to allow the local protein environment around the introduced cysteine residues to relax after mutation, especially for sites where the substitution altered side-chain polarity or packing. After this relaxation, we prepared the final conformation with phbuilder and performing the same pH ladder of the nonrelaxed conformation from pH 6 to 12 in steps of 1 pH unit, all in triplicate for 100 ns per replica.

#### DJ-1

The model of the DJ-1 dimer was based on its crystallographic structure (PDB 2OR3 ^27^). We performed the CpHMD preparation with phbuilder to the dimer. We then performed steepest-descent energy minimization (tolerance 1000 kJ mol^–1^ nm^–1^), followed by restrained NVT (1 ns) and NPT (10 ns) simulations were carried out, keeping the λ values fixed at their expected value at pH 5. All DJ-1 simulations were performed at 298 K and 100 mM NaCl, consistently with the experimental conditions used for the UV titration measurements performed in the original work.^27^ To maintain charge neutrality, 100 buffer particles have been added in the simulation box.

Finally, with the relaxed system we ran a pH ladder from pH 2 to 10 (with a step of 0.5 pH unit), and from 11 to 14 with a step of 1 pH unit; all the replicates ran for 200 ns per replica (6 replicates from 3.5 to 6, 3 replicates elsewhere), for a total of 16.2 μs.

## Results and Discussion

### Parametrization of Titratable Cysteine

Cysteine represents a nontrivial extension of constant-pH molecular dynamics because it can populate chemically distinct states in proteins. On the one hand, a free cysteine can titrate between the neutral thiol and the anionic thiolate forms. On the other hand, pairs of cysteines may form disulfide bridges, corresponding to a different covalent topology. Since the GROMACS CpHMD framework considered here is based on a fixed-topology force field, protonation changes can be described continuously within the λ-dynamics formalism, whereas bond formation and bond cleavage cannot be treated explicitly.

A first methodological choice is therefore required before introducing titratable cysteine into the workflow. In the present implementation, we restrict the constant-pH description to reduced cysteines, i.e., cysteines that remain within the thiol/thiolate form throughout the simulation. Conversely, disulfide-linked cysteines are defined *a priori* from the prepared input structure/topology and retained as standard covalent bonds during the simulation. These residues are therefore excluded from the titration scheme and treated as nontitratable disulfide-linked cysteines within the fixed bonding topology.

This choice reflects the intended scope of the method. The present implementation is not designed to describe coupled protonation–redox processes or disulfide exchange, but rather to provide a constant-pH treatment of free cysteines within a predefined redox state of the system. Consequently, the appropriateness of the selected disulfide pattern depends on prior structural and biochemical knowledge of the system and remains under user control during model preparation.

With this scope established, the next step is the introduction of a titratable cysteine residue in the modified CHARMM36 force field used by the GROMACS CpHMD implementation.

To describe reduced cysteines within this framework, we introduced a new single-site titratable residue, CYST, representing the equilibrium between the neutral thiol and anionic thiolate states,

$$\mathrm{CYS}-\mathrm{SH}⇌\mathrm{CYS}-S^{-}+H^{+}$$

The interpolation was restricted to the subset of atoms whose partial charges differ between the two protonation states, namely CB, SG, and HG1. The corresponding charge sets were taken directly from the standard CHARMM36 force field, thereby preserving the parent force-field description of cysteine and limiting the modification to the λ-dependent interpolation required by constant-pH dynamics. The intrinsic *pK*_*a*_ of cysteine is generally considered to lie in the 8–9 range depending on the chemical environment.^28^ Here, the reference *pK*_*a*_ of the model compound was set to 8.33, consistent with the commonly accepted solution value for the cysteine thiol.

This construction localizes the protonation-state change on the sulfur-containing side chain, with the largest contribution arising from the sulfur atom itself, whose partial charge changes from −0.23 in the thiol to −0.80 in the thiolate state. This shift is accompanied by a redistribution on CB, from −0.11 to −0.38, and by the disappearance of the thiol proton charge on HG1, from 0.16 to 0.00. In this way, the protonation coordinate acts on a minimal set of atoms directly involved in the titration event, consistently with the general philosophy of the original GROMACS CpHMD implementation.

Finally, the titratable definition was restricted so that only cysteines explicitly renamed as CYST during system preparation were included in the titration scheme. Standard cysteines retained as CYS, including those involved in disulfide bridges, were therefore automatically excluded from constant-pH sampling.

After defining the two protonation states of CYST, the remaining ingredient required by the CpHMD formalism is the correction potential, represented in phbuilder by the polynomial coefficients of the dvdl_1 term. This term compensates for the intrinsic force-field bias along the λ coordinate and must be calibrated for every new titratable group. Following the phbuilder parametrization protocol, we carried out this procedure on a capped ALA-CYS-ALA (ACA) tripeptide, used as a model peptide environment for cysteine. Consistently with the setup requirements, the topology of CYST was defined in its most protonated state, while the thiol/thiolate conversion was introduced through the λ-dependent interpolation of the selected atomic charges.

The ACA tripeptide (Figure 1A) was then prepared for CpHMD in calibration mode, with an initial dvdl_1 term set to zero. The system was minimized, equilibrated, and simulated at 13 fixed values of λ, from – 0.1 to 1.1 in steps of 0.1, for 1 ns each. The corresponding ⟨*dV*/*dλ*⟩_λ_ values were fitted with a fifth-degree polynomial to obtain a first estimate of the correction potential compensating the intrinsic force-field bias. This potential was then applied in a second round of minimization and equilibration, followed by ten independent 100 ns simulations at *pH* = *pK*_*a*_ = 8.33. Under these conditions, a correct correction potential should yield a uniform distribution of λ between 0 and 1. The first estimate still resulted in a nonflat λ distribution (Figure 1B), indicating residual bias. A subsequent refinement of the polynomial coefficients (the full implemented CYST is in the Supporting Information) led instead to a flat λ distribution, showing that the final correction potential successfully removed the underlying force-field preference for one protonation state over the other (Figure 1C).

**Figure 1:**
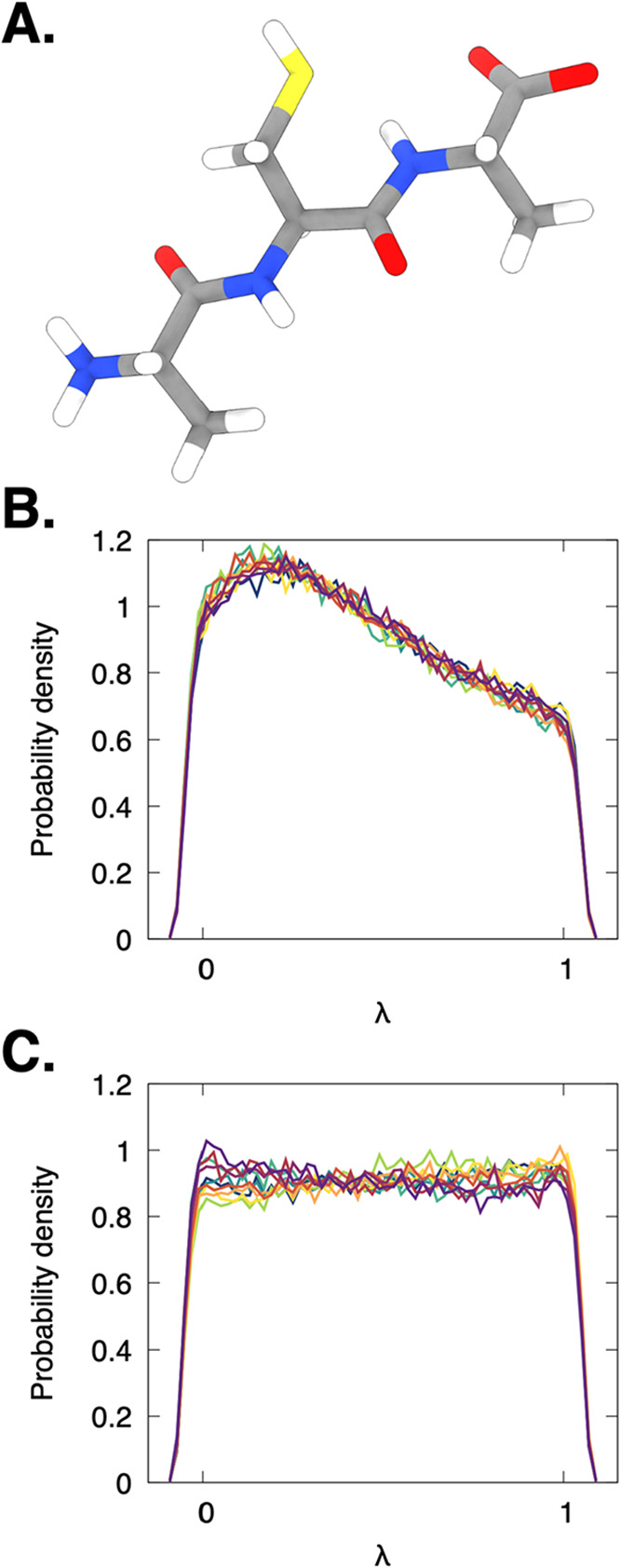
Parametrization of the CYST residue on the ACA tripeptide. (A) Structure of the ALA-CYS-ALA (ACA) tripeptide used as the model system for calibration of the cysteine correction potential. (B) Distribution of the λ coordinate obtained from ten independent 100 ns simulations at *pH* = *pK*_*a*_ = 8.33 using the first estimate of the correction potential derived from the fixed-λ calibration runs. The nonuniform distribution indicates the presence of residual force-field bias along the titration coordinate. (C) Distribution of the λ coordinate after refinement of the polynomial coefficients defining the dvdl_1 correction potential. The resulting flat distribution for λ between 0 and 1 indicates that the final correction potential effectively compensates for the intrinsic force-field bias between the thiol and thiolate states.

### ACA Tripeptide Titration

The final validation of the CYST parametrization on the model compound was performed by computing the full titration curve of the ACA tripeptide. To this end, we carried out a pH ladder from 1 to 14 with a spacing of 1 pH unit. Each pH value was simulated for 100 ns in triplicate, resulting in a total simulation time of 4.2 μs. The deprotonated fraction at each pH was obtained from the corresponding λ trajectories and averaged over the three independent replicas.

The resulting titration curve is reported in Figure 2.

**Figure 2:**
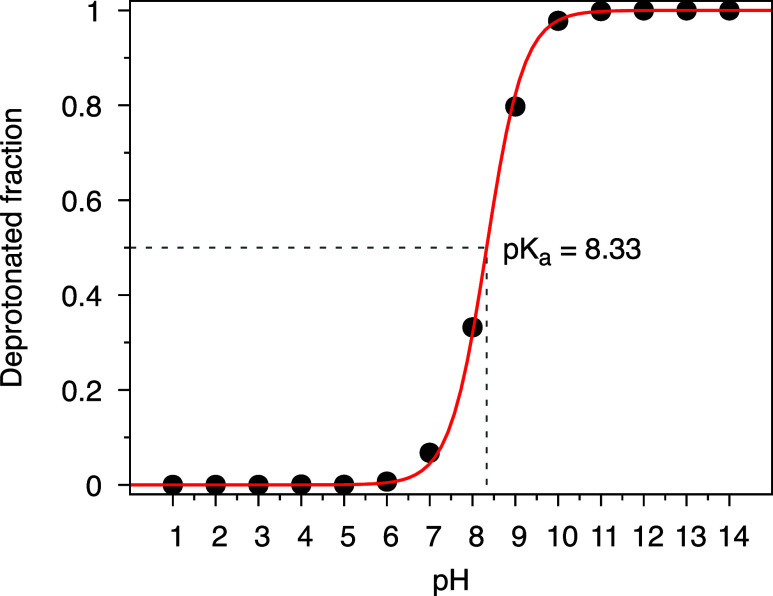
Titration curve of the ACA tripeptide. Deprotonated fraction of the CYST residue as a function of pH, obtained from CpHMD simulations of ACA. The pH ladder spans values from 1 to 14 in steps of 1 pH unit, with three 100 ns replicas for each pH point. The solid line corresponds to the Henderson–Hasselbalch fit, yielding a *pK*_*a*_ of 8.33.

The deprotonated population increases smoothly from nearly zero at acidic pH to unity at strongly basic pH, showing the expected sigmoidal behavior for a single titratable site. The data were fitted to the Henderson–Hasselbalch equation

$$f_{\mathrm{deprot}}\left(\mathrm{pH}\right)=\frac{1}{1+10^{\left(pK_{a}-\mathrm{pH}\right)}}$$

where *f*_deprot_ is the deprotonated fraction at a given pH. The fit yields a *pK*_*a*_ of 8.33 (CI 95% [8.30, 8.37]), matching the target value adopted in the calibration procedure. Therefore, the calibrated CYST residue accurately reproduces the acid–base properties of cysteine in the ACA tripeptide and confirms the consistency of the implemented correction potential.

The end point populations remain well separated along the full pH ladder, with the protonated thiol state dominating at acidic pH and the deprotonated thiolate state becoming prevalent under basic conditions.

### ACBP Titration

To test the capability of our titratable cysteine model to represent solvated residues, we focused our attention on the acyl coenzyme A binding protein (ACBP). In a previous work,^26^ this system has been studied by expressing multiple single-point mutants in cysteine and measuring the *pK*_*a*_ of all these sites (Figure 3).

**Figure 3:**
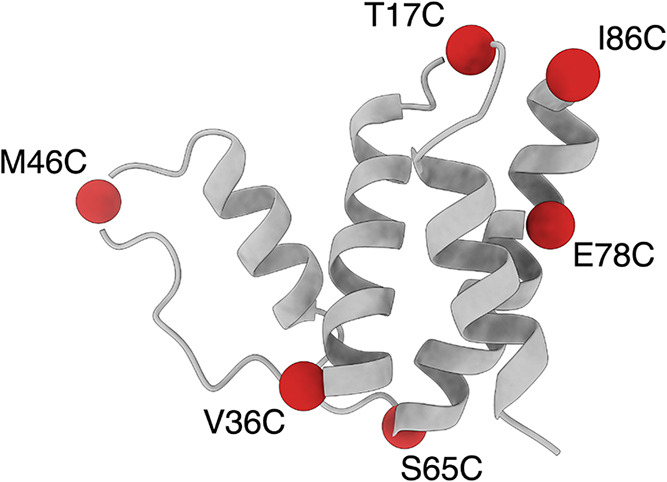
ACBP cartoon with the engineered cysteine sites (T17C, V36C, M46C, S65C, E78C, I86C) shown as spheres.

This guarantees that none of the cysteines are involved in catalytic effects, and the main factor that can influence the *pK*_*a*_ value is the solvent accessibility and the residues local interaction network. In other words, this benchmark tests whether the implementation can respond to differences in solvent exposure and local environment in the absence of catalytic ion-pair effects.

We prepared all the single-point mutants starting from the Protein Data Bank structure 1NTI,^25^ obtaining the six initial conformations of the mutants. We prepared and equilibrated the systems (see Methods), and performed a pH ladder from 6 to 12 with step 1. Each pH condition was run for 100 ns in triplicate, for a total simulation time of 2.1 μs per mutant. As in the ACA tripeptide, we then computed the protonation state population for the CYST residue and fitted with the Henderson–Hasselbalch equation. To compute the confidence interval we performed a bootstrap (see SI for the error treatment). The comparison between the experimental^26^ and predicted *pK*_*a*_ is in Figure 4A and in Table 1.

**Figure 4:**
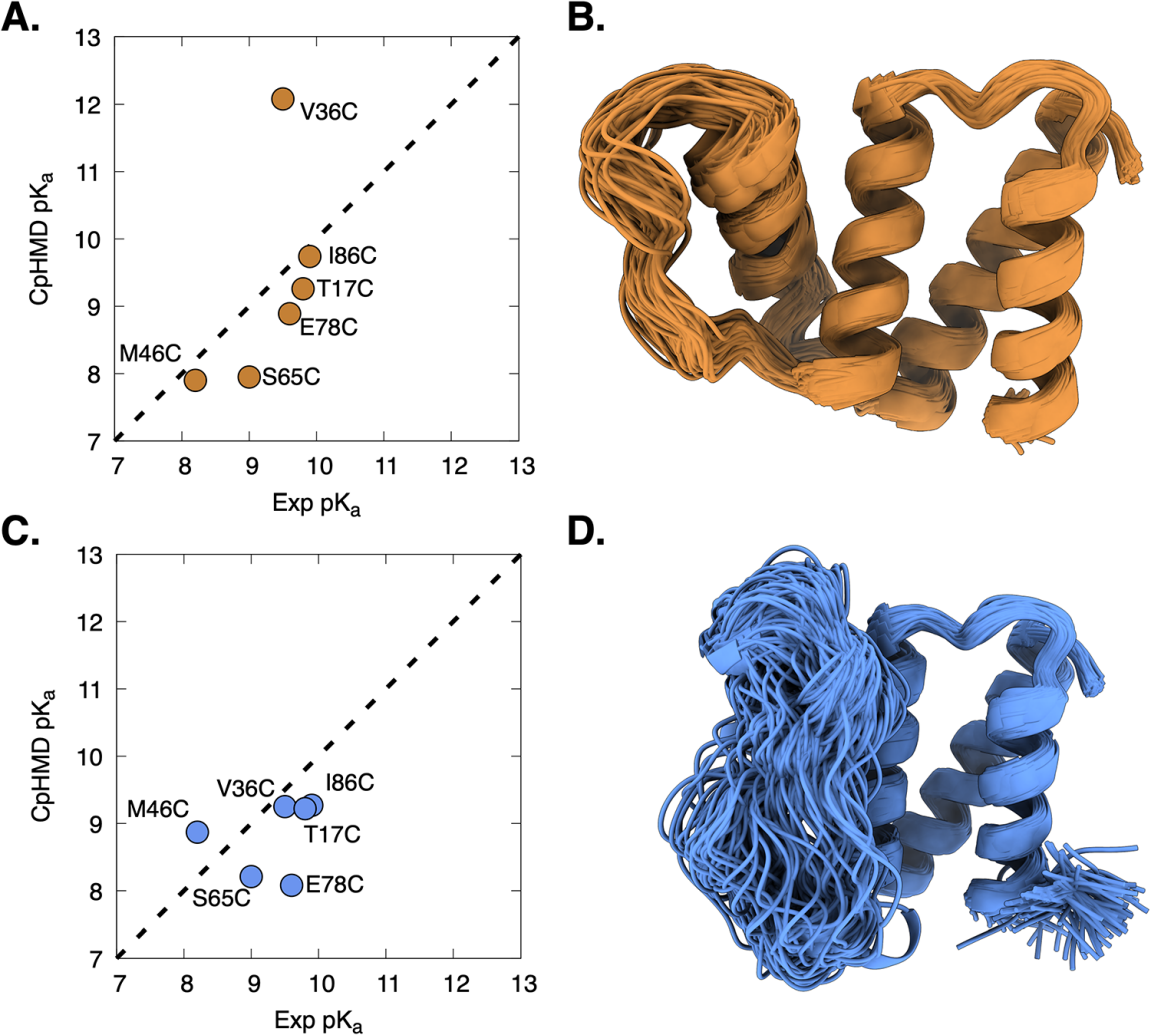
CpHMD *pK*_*a*_ benchmarking on ACBP single-cysteine mutants and rescue of the V36C outlier by structural relaxation. (A) Predicted CpHMD *pK*_*a*_ plotted against the experimental *pK*_*a*_ (RMSE 1.20); V36C deviates by >2 pH units when starting from the original (nonrelaxed) mutant structure. (B) Superposition of representative conformations of V36C at pH 10 (replica 1) from the original setup. (C) Recomputed CpHMD *pK*_*a*_ values after relaxation show improved agreement with experiment (RMSE 0.83), consistent with removal of nonequilibrated starting-state bias that primarily affected V36C. (D) Superposition of representative conformations of V36C at pH 10 (replica 1) from the setup rebuilt after a 500 ns fixed-protonation MD relaxation at pH 7: relaxation markedly changes the sampled conformational space in the region surrounding residue 36, shifting V36C from a more buried to a more solvent-exposed environment, explaining the observed difference.

**Table 1:** Experimental *pK*_*a*_ Values and Predictions with 95% Confidence Intervals for ACBP Mutants

mutant	Exp *pK*_*a*_ ^a^	CpHMD *pK*_*a*_ (not relaxed)	CpHMD *pK*_*a*_ (relaxed)
T17C	9.8 ± 0.1	9.26 [9.19, 9.32]	9.22 [9.09, 9.31]
V36C	9.5 ± 0.1	12.08 [10.86, 12.71]	9.25 [8.98, 9.74]
M46C	8.2 ± 0.1	7.90 [7.65, 8.19]	8.87 [8.47, 9.32]
S65C	9.0 ± 0.1	7.95 [7.88, 8.01]	8.21 [8.01, 8.35]
E78C	9.6 ± 0.1	8.89 [8.65, 9.11]	8.08 [8.04, 8.12]
I86C	9.9 ± 0.2	9.74 [9.49, 10.33]	9.27 [8.81, 9.95]

a From ref 26.

CpHMD-based evaluation of *pK*_*a*_ for this system yields a RMSE of 1.20 pH units, in line with the state-of-the-art in the MD-based prediction of *pK*_*a*_ ^4,6,29^ (see Table S1 in the Supporting Information). A single mutant appears as an outlier: V36C, which has a predicted *pK*_*a*_ which deviates ∼2.5 pH points from the experimental value. Observing the position of V36 (see Figure 3), and taking into account that the mutation from valine to cysteine introduces a substantially more polar side chain, we considered the possibility that the model did not have the time to relax enough to have a realistic estimate of the *pK*_*a*_. This interpretation is consistent with the limited structural relaxation observed during the CpHMD simulations started from the original V36C model (Figure 4B), suggesting that the mutation remained trapped in a nonequilibrated local environment.

We thus decided to perform a long relaxation run before the CpHMD protocol on all the mutants we prepared, allowing larger rearrangements of the portion of the protein close to the mutation site. Operatively, we set up a conventional (with the protonation states expected at pH 7) 500 ns long MD run to allow an initial equilibration of the mutants, taking the last frame as the initial frame of the CpHMD minimization-equilibration and run. This procedure reduces bias arising from nonequilibrated local environments in the starting mutant structures.

After this relaxation, the evaluation of the *pK*_*a*_ shows a dramatic improvement for V36C, and relatively small changes in all the other mutants (see Figure 4C and Table 1), returning a final RMSE of 0.83. Considering V36C, the conformational space explored in this second run is substantially different (see Figure 4D), with a broader conformational ensemble sampled by the loop containing residue 36. Notably, while the relaxation protocol strongly improves V36C, it does not uniformly improve all mutants, indicating that the remaining deviations cannot be attributed solely to starting-structure bias. Furthermore, although the agreement improves substantially after relaxation, the relative ordering of the mutants is only partially reproduced, indicating that subtle local environmental differences remain challenging to capture quantitatively.

To better characterize the physicochemical determinants that led to the change in *pK*_*a*_ estimation, we computed the distribution of the solvent accessible surface area (SASA) for the CYST side chain during the simulations at all the pH values. Regarding T17C, E78C, and I86C mutants, we obtain negligible differences in the SASA histograms, while some shift is present in M46C and S65C (see Figure S8). In agreement with the qualitative observation of the movement of V36C, the resulting distribution of SASA appears completely different: narrow and peaked in the nonrelaxed case, while extremely broad in the simulations started from the relaxed structure. This observation supports the idea that, in V36C, the apparent *pK*_*a*_ is controlled more by the breadth of the local conformational ensemble than by a single static solvent-exposure value, explaining why prior relaxation has such a strong effect on the predicted titration behavior. Regarding the other mutants we can observe a qualitative shift toward higher or lower SASA (S65C and M46C, respectively), while the others (T17C, E78C, and I86C) maintain the same SASA distribution. We thus cannot connect directly this observable to the *pK*_*a*_ estimate.

Overall, these results indicate that the CYST implementation reproduces the correct high-*pK*_*a*_ regime of noncatalytic cysteines in protein environments, while also highlighting the importance of adequate local structural equilibration and sampling for quantitatively reliable predictions.

### DJ-1 Titration

As a last test case, we want to verify the capability of the new cysteine model to correctly describe the titration curve of a catalytic cysteine. The test system is the Parkinson disease protein 7, also known as DJ-1, a homodimeric protein which contains a catalytic cysteine, Cys106 (Figure 5). This cysteine shows a marked *pK*_*a*_ shift, measured as 5.4 ± 0.1, due to the presence of a second residue, GLU18, which appears always protonated.^27^

**Figure 5:**
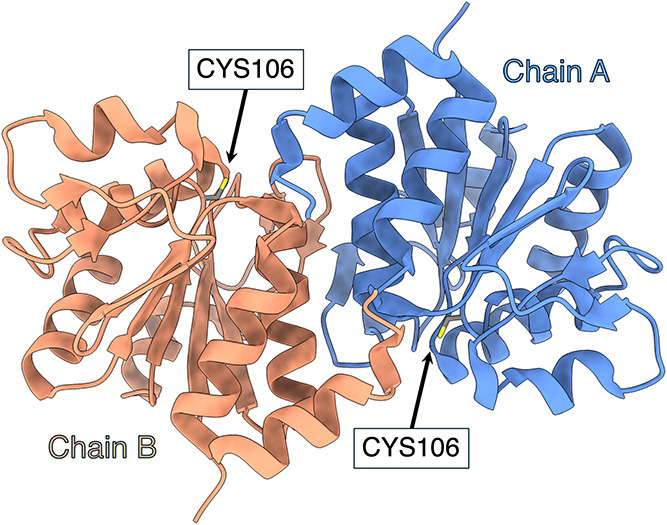
Cartoon representation of the homodimeric DJ-1 protein, with chain A shown in blue and chain B in salmon. The two reactive cysteines, Cys106, are highlighted in yellow and indicated by arrows. Both copies of Cys106 were monitored in the CpHMD simulations and analyzed as chain-specific observations of the same reactive site.

Here we verify the ability of the CYST implementation to describe properly the catalytic cysteine coordinated with the protonation state of connected acidic residues.

Because DJ-1 is a symmetric homodimer, both Cys106 residues in chains A and B should in principle display the same titration behavior at convergence, providing an internal consistency check for the simulations.

We prepared and equilibrated the model of DJ-1 (see Methods), and performed a pH ladder from 2 to 10 with step 0.5, and from 11 to 14 with step 1. Each pH condition was run for 200 ns in triplicate (and in sextuplicate for the Cys106 transition pH range, from 3.5 to 6.0), for a total simulation time of 16.2 *μs*. As in the previous cases, we then computed the protonation state population for the Cys106 residues in both chains and fitted them with the Henderson–Hasselbalch equation (details on the error treatment in the Supporting Information).

As expected for a reactive cysteine, both Cys106 residues titrate in a markedly more acidic pH range than the model peptide and the noncatalytic ACBP mutants. The chain-specific Henderson–Hasselbalch fits yield apparent *pK*_*a*_ values of 3.94 (95% CI: [3.67,4.19]) for chain A and 4.77 (95% CI: [4.30,5.20]) for chain B (Figure 6B), with the latter in reasonable agreement with the experimental value of 5.4 ± 0.1. Despite the protomer-specific asymmetry, the DJ-1 calculations place the titration of Cys106 in the correct acidic regime, suggesting that the CYST implementation captures strong acidification in DJ-1. We compare our results with the state-of-the-art in Table S2 in the Supporting Information.

**Figure 6:**
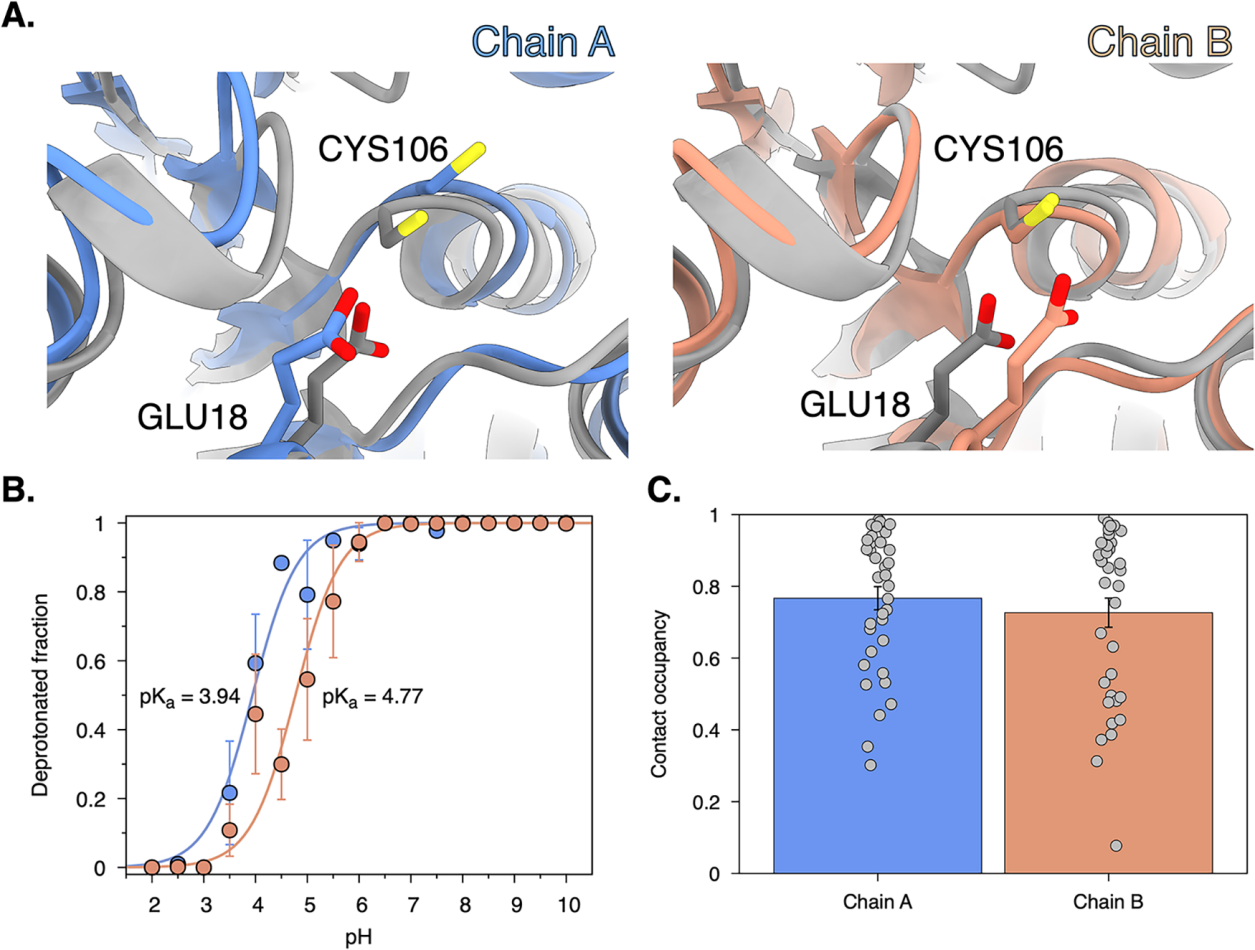
Protomer-specific titration behavior of Cys106 in DJ-1 and analysis of the chain asymmetry. (A) Representative structures after equilibration showing the local arrangement of Cys106 and Glu18 in chain A (blue) and chain B (salmon). Cys106 and Glu18 are shown as sticks. (B) Chain-specific titration curves of Cys106 obtained from CpHMD simulations and fitted to the Henderson–Hasselbalch equation, yielding apparent *pK*_*a*_ values of 3.94 for chain A and 4.77 for chain B. While chain B is close to the experimental value, chain A remains shifted toward lower *pK*_*a*_. (C) Occupancy of short (<4 Å) Cys106-Glu18 contacts, pooled over the pH range spanning the titration transition (from 3.5 to 6.0), for chain A and chain B. Despite the difference in apparent *pK*_*a*_, the two chains display similar contact occupancies, indicating that the protomer asymmetry cannot be explained solely by a simple difference in Cys106-Glu18 proximity.

At the same time, the two protomers do not converge to identical titration behavior within the present simulation time, even though they are symmetry-related in the experimental structure. Inspection of the equilibrated conformations shows that the local arrangement of Cys106 and Glu18 differs between the two chains (Figure 6A), suggesting a possible origin for the different apparent *pK*_*a*_ values. To test this hypothesis more directly, we quantified the occupancy of short Cys106-Glu18 contacts over the pH range spanning the titration transition, pooling the data from pH 3.5 to 6.0. However, the resulting occupancies are very similar in the two chains (Figure 6C), indicating that the protomer-specific asymmetry cannot be explained solely by a simple difference in Cys106-Glu18 proximity.

We also examined additional local descriptors of the Cys106 environment, including Glu18 protonation behavior (Figure S13), solvent exposure of Cys106 (Figure S11), side-chain conformational preferences (Figure S12), and Arg28/Arg48-anion minimum distance distribution (Figure S14), as suggested by experimental^27^ and computational^30^ works, but none of these quantities alone provided a clear explanation for the observed difference between the two protomers. Analyzing the titration behavior of Glu18, it remains protonated throughout the Cys106 titration range in both protomers, with a strongly shifted apparent *pK*_*a*_ of 10.84 (CI 95% [10.52, 11.50]) for chain A and 10.34 (CI 95% [10.02, 10.53]) for chain B. This result is qualitatively consistent with previous experimental work, where Glu18 was considered protonated over the Cys106 titration range,^27^ and with computational work in which Glu18 was predicted to have a very high *pK*_*a*_ (>14) when Cys106 is deprotonated.^30^ However, because Glu18 displays a high apparent *pK*_*a*_ in both chains, its protonation behavior does not by itself explain the different apparent *pK*_*a*_ values obtained for Cys106 in the two monomers. Similarly, solvent exposure and side chain conformational preferences did not provide a clear explanation for the observed difference between the two protomers. We therefore interpret the chain-specific shift in apparent *pK*_*a*_ as arising either from subtler local electrostatic effects not captured by these simple observables or from residual finite-sampling effects. In this sense, DJ-1 represents a more demanding benchmark than ACA tripeptide or ACBP: it confirms that the present CYST implementation can reproduce the strongly shifted low-*pK*_*a*_ regime of a reactive cysteine, while also showing that quantitative convergence and mechanistic resolution remain more challenging in coupled protein environments.

## Conclusions

In this work, we implemented a titratable cysteine residue, CYST, within the GROMACS-based λ-dynamics constant-pH molecular dynamics framework and integrated it into the phbuilder workflow. The new residue describes the thiol/thiolate equilibrium of reduced cysteines within a fixed-topology force field, while disulfide-linked cysteines are retained as nontitratable covalent states defined *a priori*. This choice reflects the intended scope of the CYST implementation: to model cysteine protonation equilibria in a predefined redox topology, rather than coupled protonation-redox transitions.

Calibration on the ACA tripeptide shows that the final correction potential successfully removes the intrinsic force-field bias along the λ coordinate and reproduces the target cysteine titration behavior, yielding a *pK*_*a*_ of 8.33 for the model compound. The corresponding end point populations remain well separated across the full pH ladder, indicating that the implemented CYST residue correctly captures the intrinsic thiol/thiolate balance of cysteine in a peptide environment.

Application to six engineered single-cysteine mutants of ACBP further shows that the implementation reproduces the correct high-*pK*_*a*_ regime expected for noncatalytic protein cysteines. In this benchmark, the largest error was traced to a nonequilibrated local environment in the V36C mutant, and a preliminary conventional MD relaxation substantially improved the agreement with experiment. At the same time, the ACBP results also indicate that quantitative prediction remains sensitive to the underlying conformational ensemble and that local structural equilibration is an essential prerequisite for reliable protein *pK*_*a*_ estimation.

Finally, the DJ-1 test case demonstrates that the present implementation can capture the strongly shifted low-*pK*_*a*_ regime of a reactive cysteine in a protein environment. Although the two protomers did not converge to identical titration behavior within the present simulation time, one copy of Cys106 yielded an apparent *pK*_*a*_ close to the experimental value, placing the titration in the correct acidic regime overall. The remaining protomer-specific asymmetry could not be straightforwardly rationalized by simple geometric or solvent-exposure descriptors, suggesting that more subtle electrostatic effects or residual finite-sampling limitations are still at play in this more challenging benchmark.

The present results also indicate where further methodological developments should be directed. In ACBP V36C and in DJ-1, the limiting factor is not only the sampling of the protonation coordinate, but also the sampling of the structural and electrostatic environment coupled to it. Enhanced sampling strategies could therefore be useful in future applications. Replica-exchange or REST2-like approaches could accelerate local side chain and loop rearrangements, whereas metadynamics or similar biased sampling schemes could target descriptors such as cysteine solvent exposure, Cys-acidic-residue distances, local hydration, or salt/ion occupancy. Such approaches may reduce the dependence on the initial conformational ensemble and improve convergence in coupled protein environments.

Taken together, these results establish the present CYST implementation as a practical extension of the GROMACS CpHMD framework for the study of reduced cysteine protonation equilibria in peptides and proteins. More broadly, they provide a basis for future applications to reactive cysteines in catalytic sites, ligandable cysteines in covalent drug discovery, and pH-dependent cysteine chemistry in biomolecular systems. At the same time, the accuracy of cysteine *pK*_*a*_ predictions is expected to remain limited by the underlying fixed-charge force field and by the treatment of the sulfur chemical environment. Cysteine *pK*_*a*_ values are known to be particularly challenging to predict compared with more conventional titratable residues such as Asp, Glu, or Lys, in part because the polarizability and specific nonbonded interactions of the thiol/thiolate group are difficult to represent in standard additive force fields.^31^ Future developments should aim at improving sampling of coupled protonation/conformational states in protein environments and at refining the physical description of the cysteine side chain, for example through charge-scaling strategies,^29^ targeted nonbonded corrections such as NBFIX terms, or polarizable force-field formulations such as Drude models.^32^ Such developments would be complementary to the present implementation, which focuses on introducing a working fixed-charge titratable cysteine residue within the existing GROMACS CpHMD framework.

## Supplementary Material



## Data Availability

All input files required to reproduce these results, together with the trajectories of the runs shown here, are available on the Zenodo repository with the DOI 10.5281/zenodo.19339340. The modified phbuilder version which contains the CYST residue and the preparation script for cysteines is available at https://gitlab.com/riccardocapelli/phbuilder.

## Notes

CpHMD parameters for the CYST residue; extended description of end point-state assignment, bootstrap resampling, and confidence-interval calculation for *pK*_*a*_ estimates; comparison of cysteine *pK*_*a*_ predictions with external computational approaches; λ-coordinate distributions for the ACBP cysteine mutants before and after structural relaxation; time-dependent *pK*_*a*_ convergence analysis for the ACBP mutants before and after relaxation; cysteine SASA distributions for relaxed and nonrelaxed ACBP mutants; deprotonated-fraction curves for DJ-1 Glu18 in chains A and B; time-dependent *pK*_*a*_ convergence analysis for DJ-1 Cys106; Cys106 SASA distributions for DJ-1 chains A and B; Cys106 χ_1_ distributions for DJ-1 chains A and B; and chloride-arginine minimum-distance distributions for DJ-1 chains A and B (PDF)
