# Supplementary material for: Implementation and Validation of Titratable Cysteine in GROMACS-Based Constant-pH Molecular Dynamics
Source: J Chem Theory Comput. 2026 Jun 8;22(12):6128–36. doi: 10.1021/acs.jctc.6c00640 (PMC13296518; doi:10.1021/acs.jctc.6c00640)
Supplement: Supplementary file 1 [file ct6c00640_si_001.pdf]

Supporting information for: "Implementation and Validation of Titratable Cysteine in GROMACS-based Constant-pH Molecular Dynamics"

Riccardo Capelli*

Department of Biosciences, Università degli Studi di Milano, Via Celoria 26, I-20133 Milan, Italy

E-mail: riccardo.capelli@unimi.it

CpHMD parameters of CYST

```
[ CYST ]
incl = CYST
atoms = CB SG HG1
qqA = -0.11  -0.23  0.16
pKa_1 = 8.33
qqB_1 = -0.38  -0.80  0.00
dvdl_1 = 80.068 -90.98 125.628 -272.829 -519.705 -25.106
```

Error treatment

For all CpHMD trajectories, protonation-state populations were extracted from the corresponding λ trajectories using endpoint counting. After discarding the first 50% of each trajectory as equilibration, frames with $\lambda < 0.2$ were assigned to the protonated state and frames with $\lambda > 0.8$ to the deprotonated state, while intermediate frames were not included in the endpoint population count. At each pH value, the deprotonated fraction was computed by pooling endpoint counts over the three replicas,

$$p_{\text{deprot}} = \frac{\sum_r N_{B,r}}{\sum_r (N_{A,r} + N_{B,r})},$$

where $N_{A,r}$ and $N_{B,r}$ are the numbers of protonated and deprotonated endpoint frames in replica r, respectively.

The uncertainty reported for the deprotonated fraction at fixed pH was obtained by bootstrap resampling over replicas. Briefly, for each pH value, the three replicas were resampled with replacement 2×10^4 times, and for each bootstrap sample the pooled deprotonated fraction was recomputed from the corresponding endpoint counts. The uncertainty reported by the analysis script is the standard deviation of the resulting bootstrap distribution, here denoted as SEM_{boot}. In addition, the analysis script outputs the deprotonated fractions of the individual replicas and the fraction of frames falling in the intermediate region $0.2 < \lambda < 0.8$, which was used as a diagnostic of incomplete endpoint separation but not directly propagated into the pK_a uncertainty.

The pK_a values were obtained by nonlinear least-squares fitting of the pH-dependent deprotonated fractions. For standard titration curves, the data were fitted to the Henderson–Hasselbalch equation,

$$f_{\text{deprot}}(pH) = \frac{1}{1 + 10^{(pK_a - pH)}}.$$

To estimate the uncertainty on pK_a, we performed bootstrap resampling over replicas consistently with the procedure used for the deprotonated fractions. For each bootstrap sample,

the replicas at every pH value were resampled with replacement, the pooled deprotonated fractions were recomputed from the corresponding endpoint counts, and the resulting titration curve was refitted to the Henderson-Hasselbalch equation. Repeating this procedure 10^4 times yielded a bootstrap distribution of fitted pK_a values. The reported pK_a uncertainty was then expressed as the 95% confidence interval, defined by the 2.5th and 97.5th percentiles of the bootstrap distribution. Therefore, the error bars shown on titration curves reflect the bootstrap uncertainty of the deprotonated fraction at each pH, whereas the reported pK_a uncertainty reflects the confidence interval of the fitted parameter obtained from bootstrap resampling.

Convergence of fitted pK_a values

To assess the robustness of the fitted pK_a values, we performed a cumulative block analysis as a function of trajectory length. After discarding the initial equilibration portion of each trajectory, endpoint populations were recomputed using progressively longer trajectory segments, and the Henderson-Hasselbalch fit was repeated for each cumulative time window. The resulting time dependence of the fitted pK_a was used to evaluate whether the reported values were stable over the simulated trajectory length.

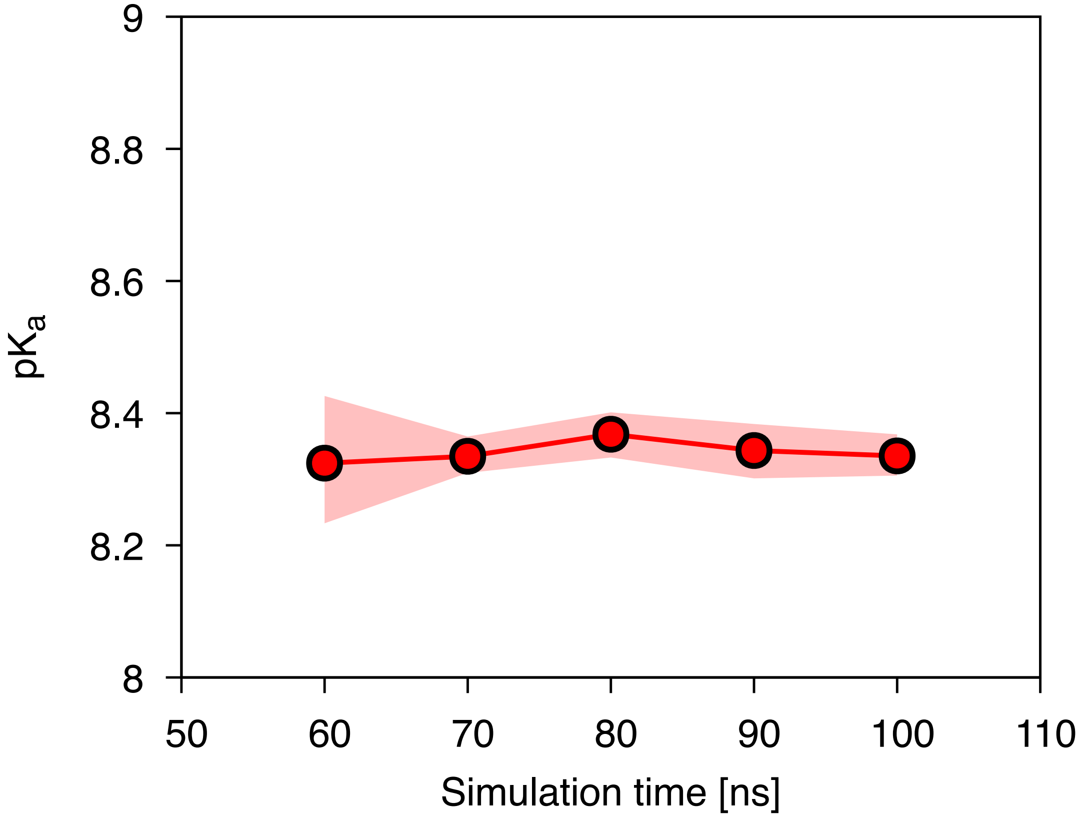

Figure S1: Cumulative pK_a estimates for the ACA tripeptide obtained by progressively increasing the analyzed trajectory length after equilibration. Points report the bootstrap mean pK_a, and shaded regions indicate the corresponding 95% confidence intervals.

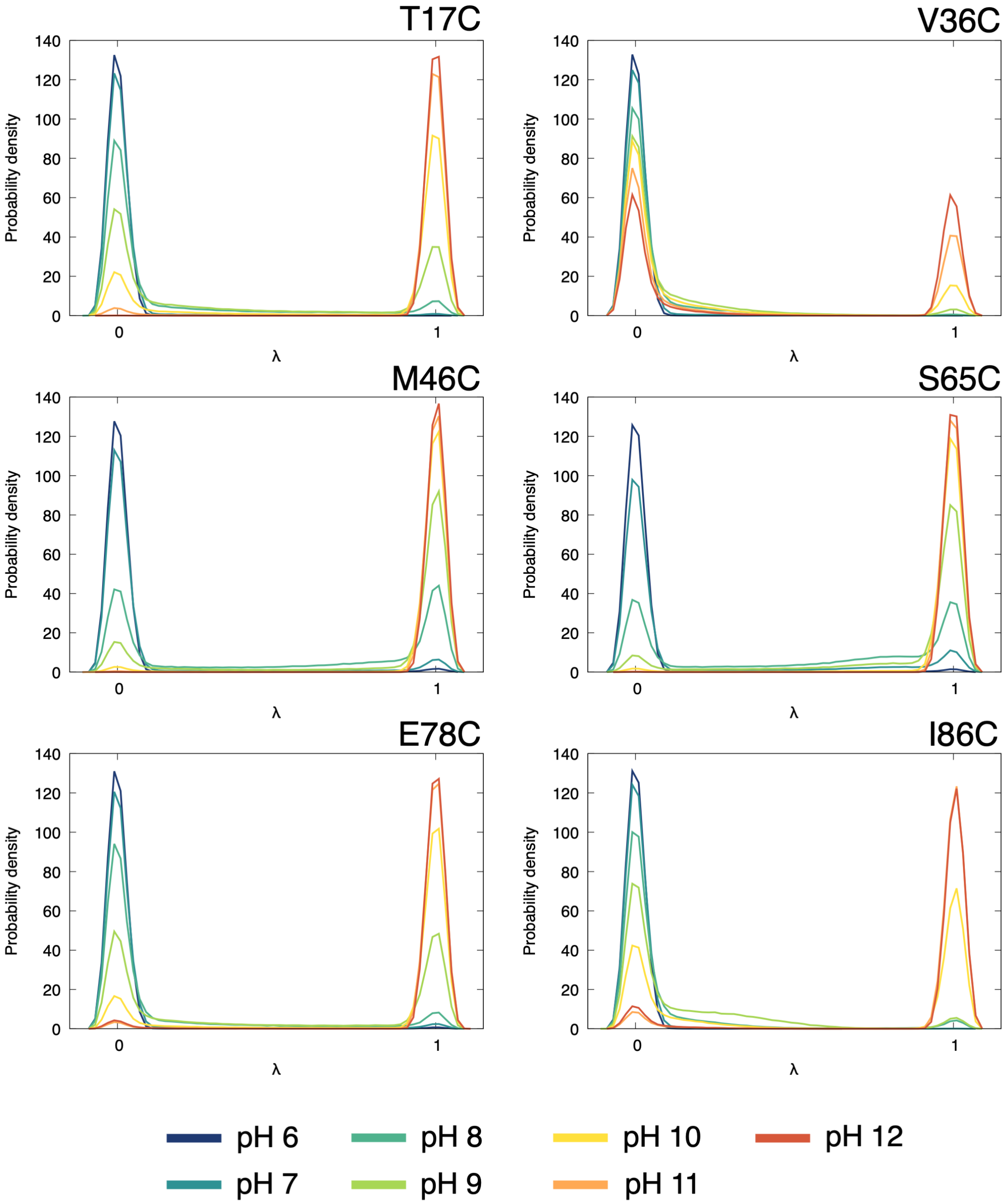

Figure S2: λ-coordinate distributions of the titratable cysteine residue in the ACBP single-cysteine mutants before structural relaxation. Each panel reports one mutant (T17C, V36C, M46C, S65C, E78C, and I86C). Distributions were obtained by pooling the three independent replicas at each pH value. The curves show the expected pH-dependent redistribution of CYST from the protonated endpoint ($\lambda \simeq 0$) at low pH to the deprotonated endpoint ($\lambda \simeq 1$) at high pH, with mixed endpoint populations in the transition region. The small population of V36C at $\lambda = 1$ is evident.

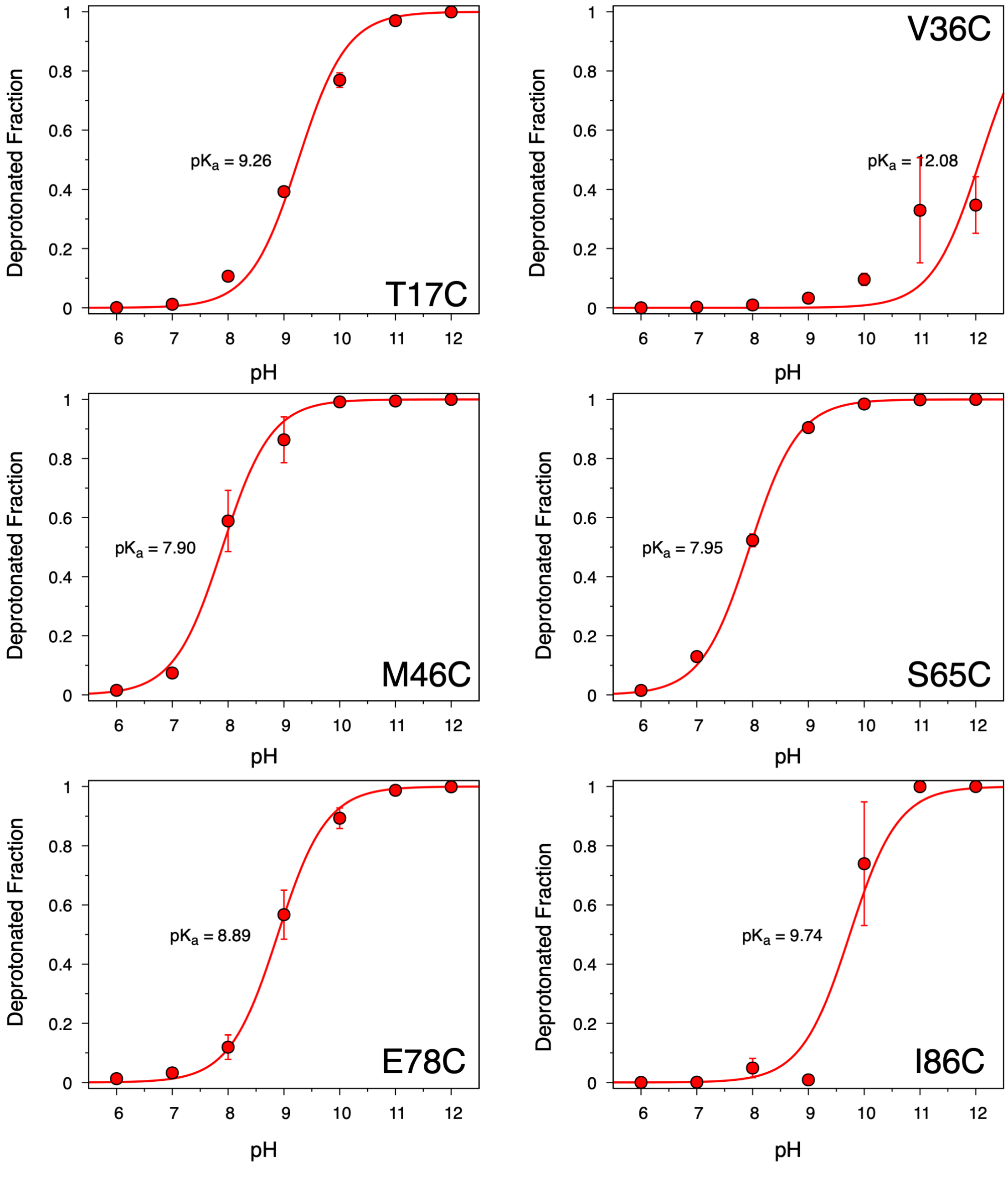

Figure S3: Titration curves for cysteine residue in the ACBP single-cysteine mutants before structural relaxation. The pH-dependent behavior has been fitted to Henderson-Hasselbalch (continuous line).

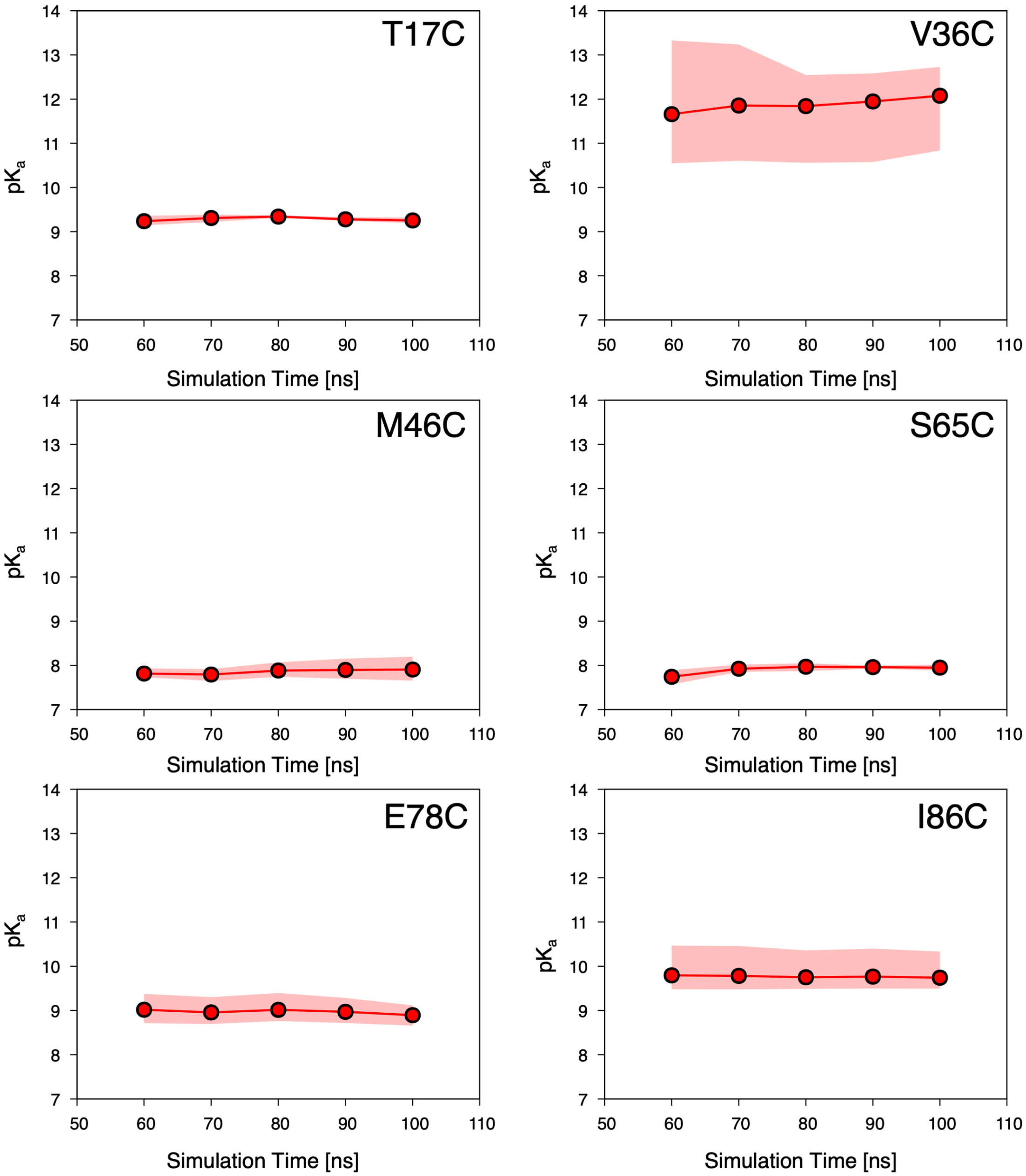

Figure S4: Cumulative pK_a estimates for the six ACBP single-cysteine mutants obtained by progressively increasing the analyzed trajectory length after equilibration. Points report the bootstrap mean pK_a, and shaded regions indicate the corresponding 95% confidence intervals. The analysis shows stable estimates for most mutants, whereas V36C and I86C display broader confidence intervals. For V36C, this behavior is attributed to the non-relaxed starting structure, whereas for I86C it is associated with its C-terminal position and the resulting larger conformational space.

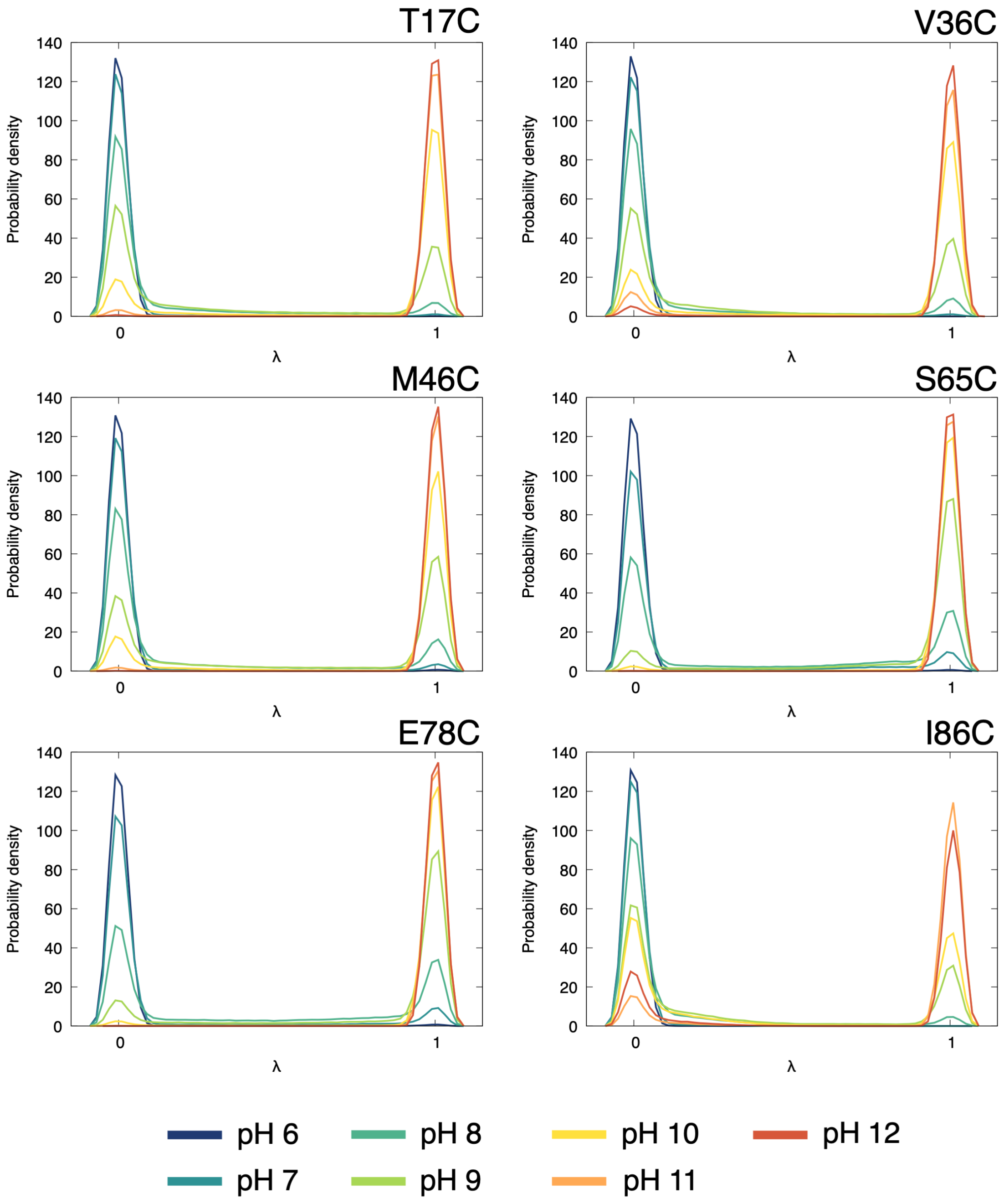

Figure S5: λ-coordinate distributions of the titratable cysteine residue in the ACBP single-cysteine mutants after the 500-ns fixed-protonation structural relaxation. Each panel reports one mutant (T17C, V36C, M46C, S65C, E78C, and I86C). Distributions were obtained by pooling the three independent replicas at each pH value. The endpoint populations remain well separated over the pH ladder, supporting the use of endpoint counting for estimating the deprotonated fraction and fitting the cysteine titration curves. Here the λ population of V36C is more similar to the other mutants, which shifts its cysteine pK_a to a value closer to the others.

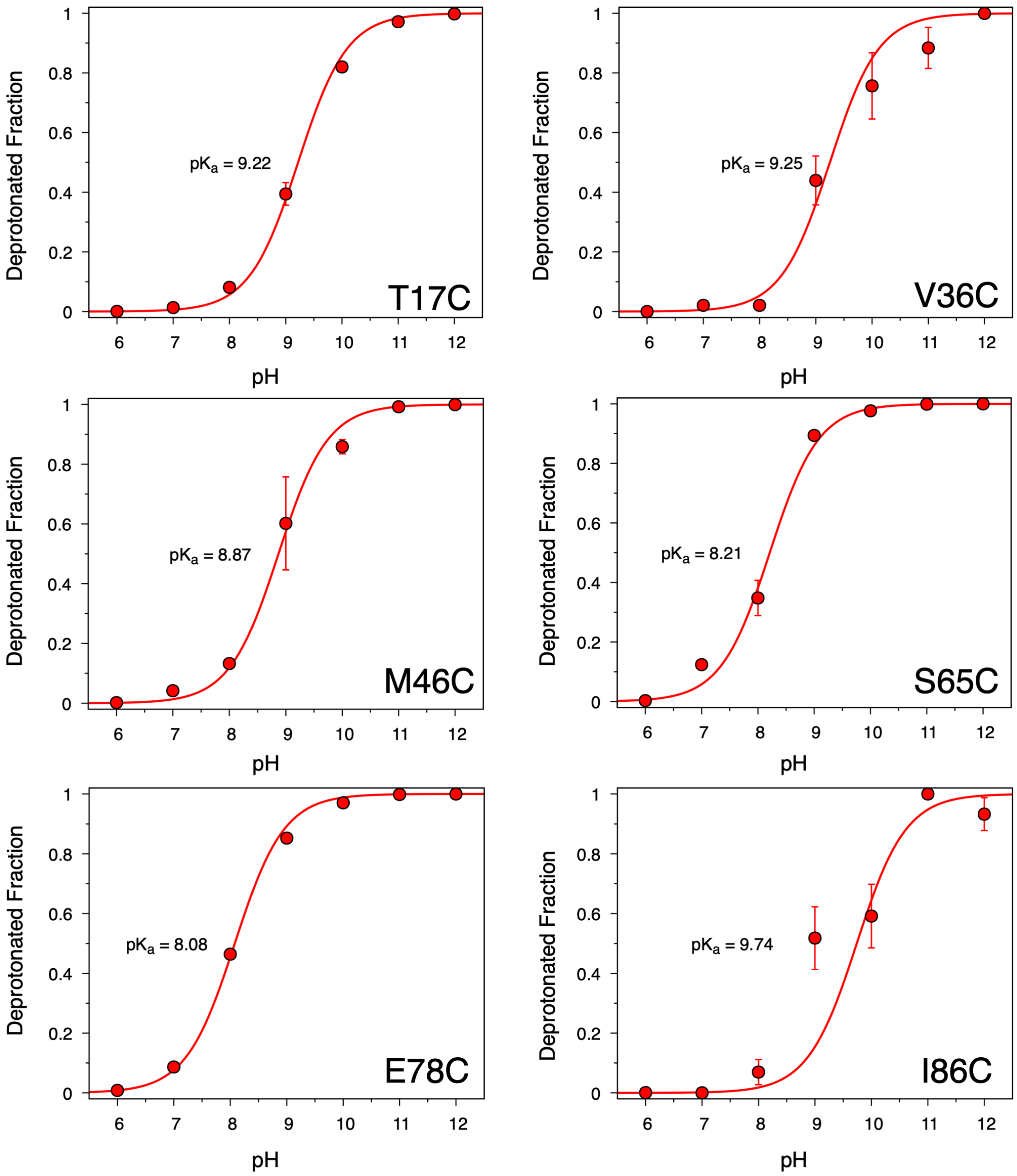

Figure S6: Titration curves for cysteine residue in the ACBP single-cysteine mutants after structural relaxation. The pH-dependent behavior has been fitted to Henderson-Hasselbalch (continuous line).

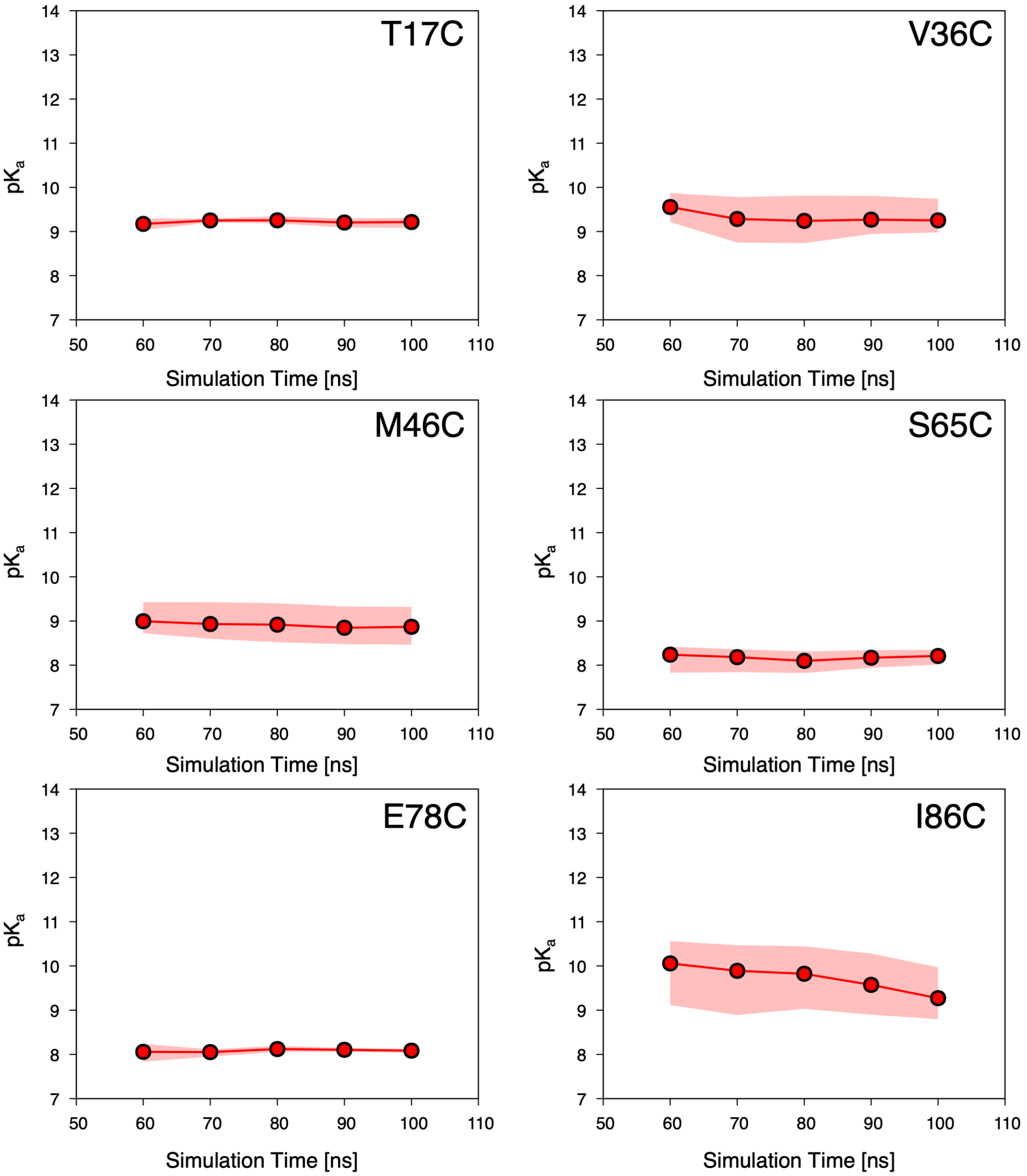

Figure S7: Cumulative pK_a estimates for the six ACBP single-cysteine mutants after rebuilding the CpHMD ladders from structures obtained following 500 ns of conventional MD relaxation. Points report the bootstrap mean pK_a, and shaded regions indicate the corresponding 95% confidence intervals. After relaxation, most mutants show stable pK_a estimates over the analyzed time window, supporting convergence of the endpoint populations; V36C remains associated with a broader uncertainty, consistent with its position in the structure.

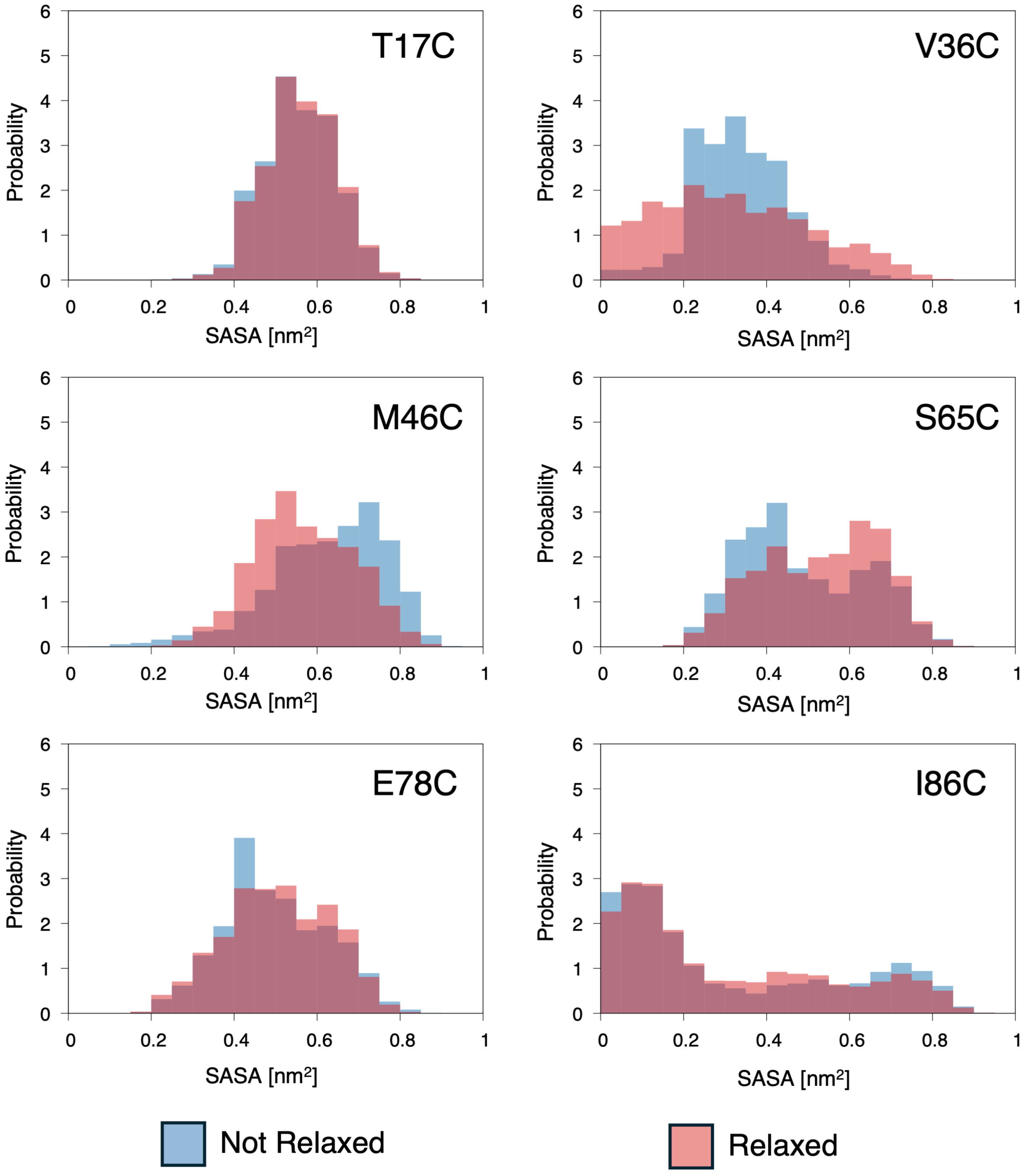

Figure S8: Distribution of cysteine solvent accessibility in the ACBP mutant set. Probability distributions of the cysteine side-chain solvent-accessible surface area (SASA) obtained by pooling all sampled SASA values over the full pH ladder for each of the six ACBP mutants. For each mutant, the distributions corresponding to the initial structures (*Not Relaxed*, blue) and to the systems after preliminary conventional MD relaxation (*Relaxed*, red) are compared. The figure highlights that the effect of the relaxation protocol on the sampled cysteine environment is modest for most mutants, whereas more noticeable redistribution of the accessible conformational ensemble is observed for selected cases, most prominently V36C.

Table S1: Comparison of experimental cysteine pK_a values with the present CpHMD calculations and previously reported predictions from Wilson et al.[1] for the ACBP mutant set. The I86C mutant was included in the present ACBP benchmark but was not present in the Wilson et al. table.

Mutant	Exp.	This work unrelaxed	This work relaxed	C36m	mC36m	ff14SB	mff14SB	PROPKA	PypKa
T17C	9.8	9.26	9.22	9.68	9.47	10.08	9.73	9.40	9.30
V36C	9.5	12.08	9.25	10.15	9.16	9.87	9.60	9.20	8.90
M46C	8.2	7.90	8.87	8.17	8.39	7.74	8.26	9.20	8.80
S65C	9.0	7.95	8.21	8.04	7.83	8.58	8.96	9.80	9.20
E78C	9.6	8.89	8.08	8.14	8.52	8.87	9.04	8.90	8.20
I86C	9.9	9.74	9.27	–	–	–	–	–	–
RMSE[a]	–	1.31	0.87	0.84	0.75	0.48	0.26	0.69	0.77

[a]RMSE values were computed only over the five ACBP mutants common to the present work and the Wilson et al. data set: T17C, V36C, M46C, S65C, and E78C.

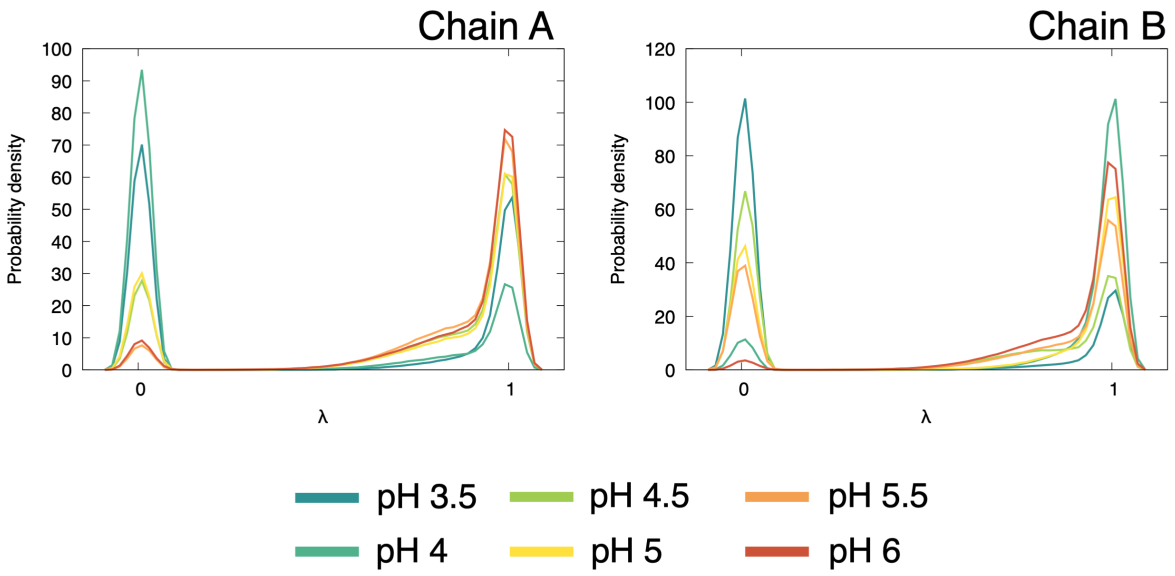

Figure S9: λ-coordinate distributions of the CYS106 residue in both chains of the DJ-1 dimer. Distributions were obtained by pooling the three independent replicas at each pH value.

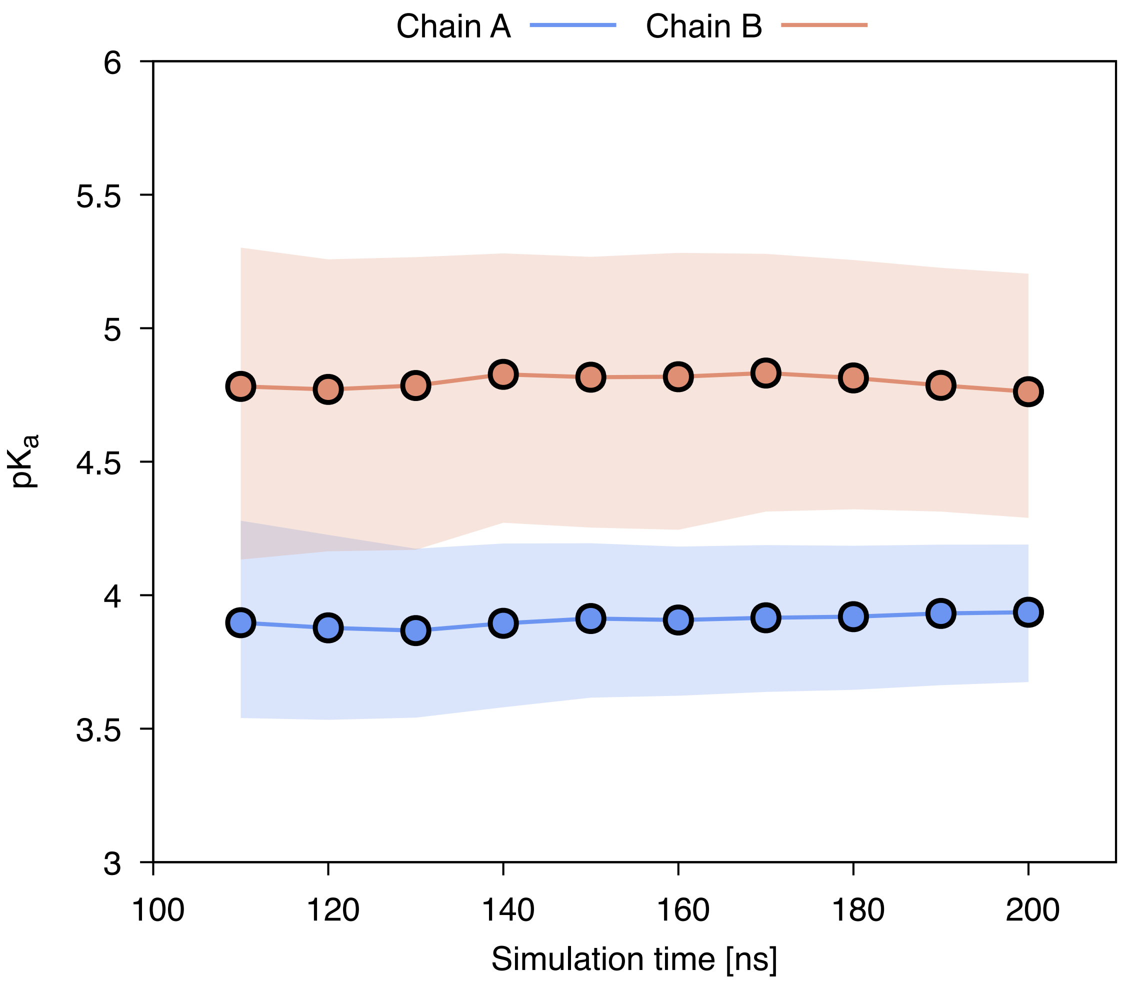

Figure S10: Time-dependent convergence of the apparent pK_a of DJ-1 Cys106 in the two protomers. The pK_a was recomputed from cumulative trajectory windows after the initial equilibration discard, pooling endpoint populations across the available replicas at each pH and refitting the Henderson–Hasselbalch equation. Solid lines report the bootstrap mean, while shaded regions indicate the 95% confidence interval. Chain A and chain B converge to distinct apparent pK_a values, confirming that the protomer-specific difference is not removed by extending the cumulative sampling window.

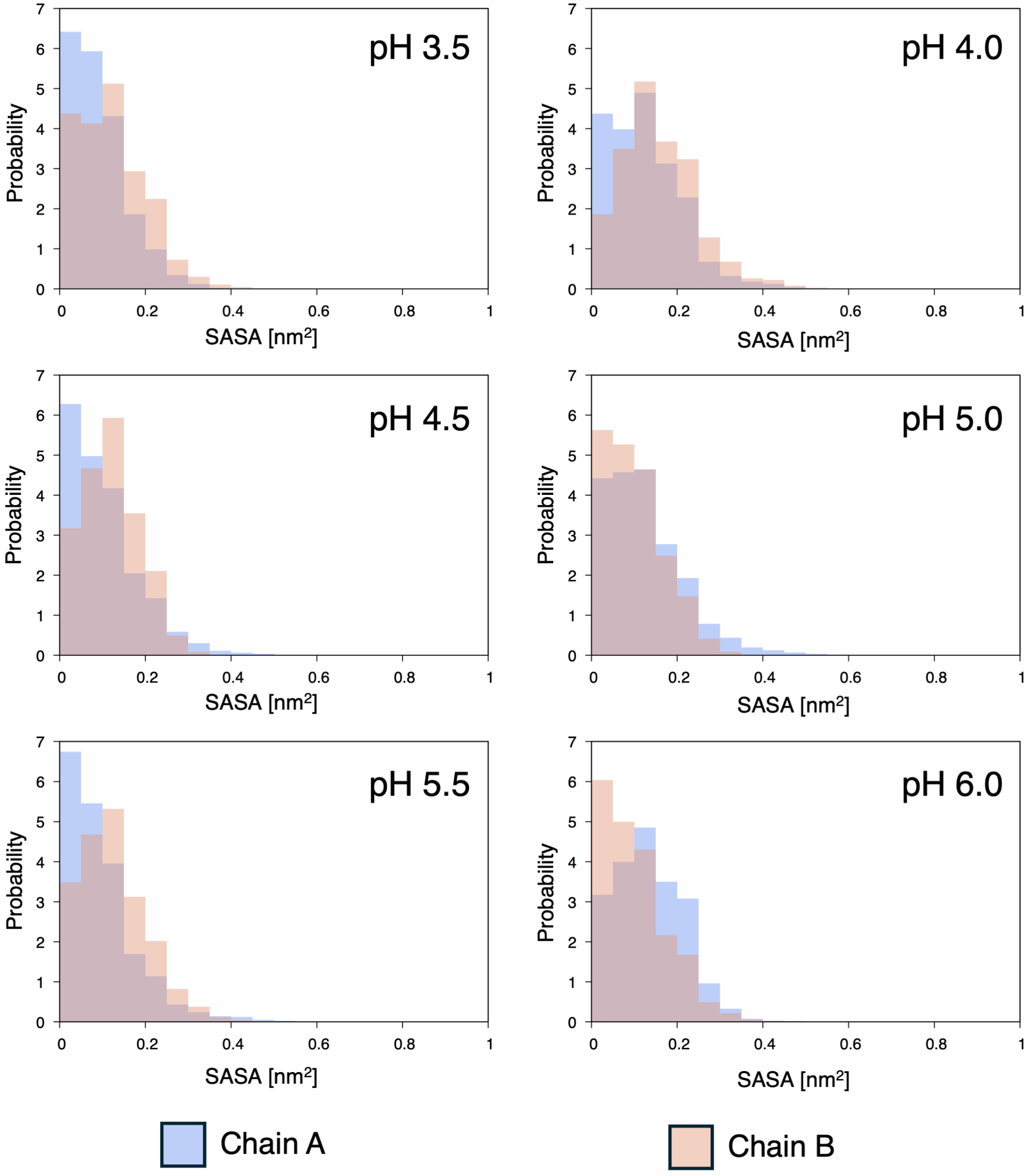

Figure S11: Probability distributions of the Cys106 side-chain solvent-accessible surface area (SASA) for chain A (blue) and chain B (salmon) of the DJ-1 dimer at selected pH values spanning the titration transition. The two protomers display similar SASA distributions across the explored pH range, with only modest chain-specific differences in solvent exposure. These results indicate that the protomer asymmetry observed in the apparent pK_a values cannot be straightforwardly explained by average differences in Cys106 solvent accessibility alone.

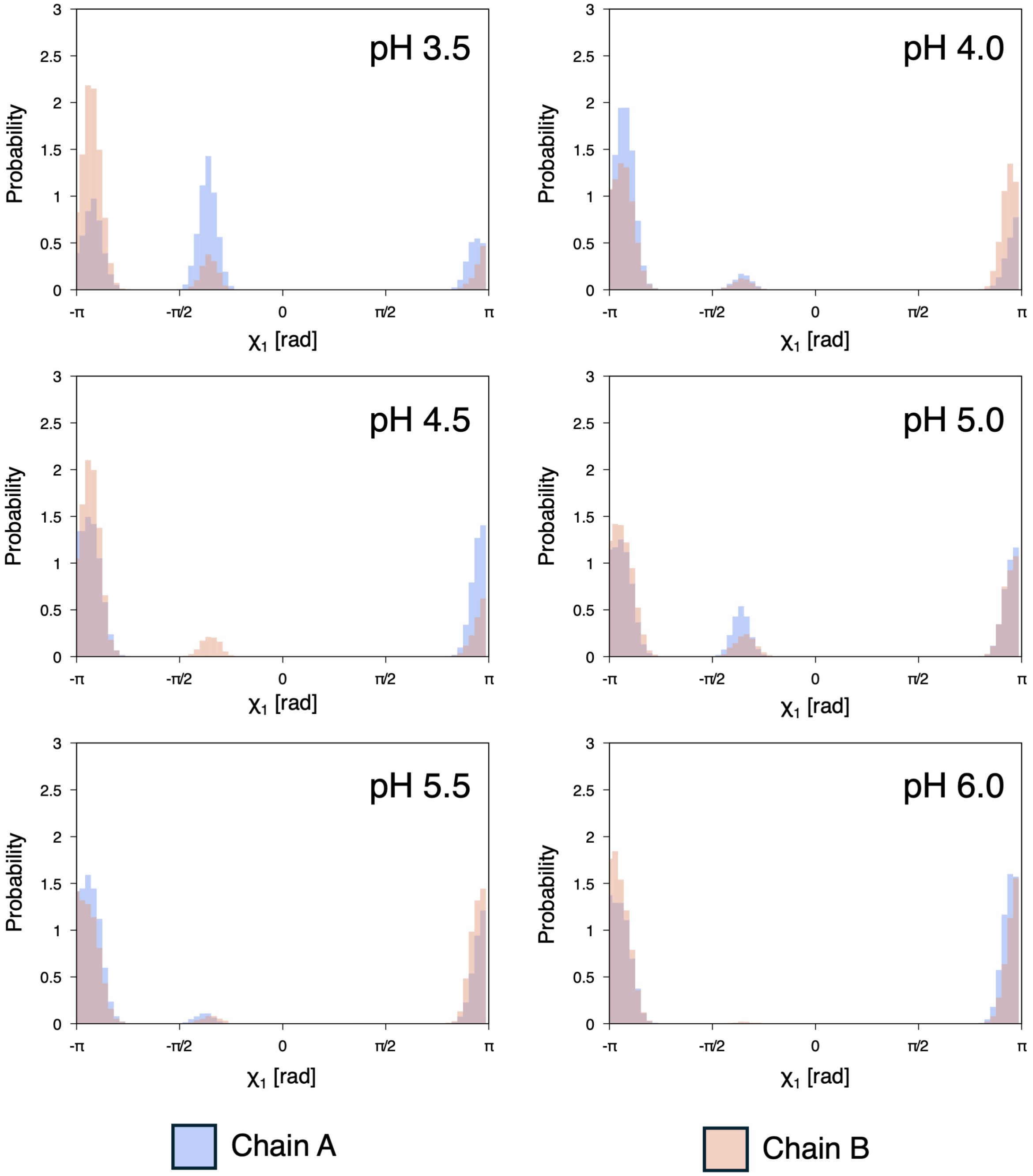

Figure S12: Probability distributions of the χ_1 dihedral angle of Cys106 for chain A (blue) and chain B (salmon) of the DJ-1 dimer at selected pH values spanning the titration transition. The two protomers populate broadly similar rotameric states across the explored pH range, with the main populations located near the same χ_1 basins and only modest differences in their relative weights. These results indicate that the protomer asymmetry observed in the apparent pK_a values cannot be straightforwardly explained by a simple difference in the side-chain conformational preferences of Cys106 alone.

Table S2: Comparison of the experimental pK_a of DJ-1 Cys106 with the present CpHMD calculations and previously reported predictions from Wilson et al.[1] The two protomers were fitted separately in the present work.

System/site	Exp.	This work	C36m	mC36m	ff14SB	mff14SB	PROPKA	PypKa
DJ-1 Cys106, chain A	5.4	3.93 [3.67, 4.17]	6.71	11.26	7.63	4.98	12.30	10.40
DJ-1 Cys106, chain B	5.4	4.82 [4.36, 5.23]	6.71	11.26	7.63	4.98	12.30	10.40

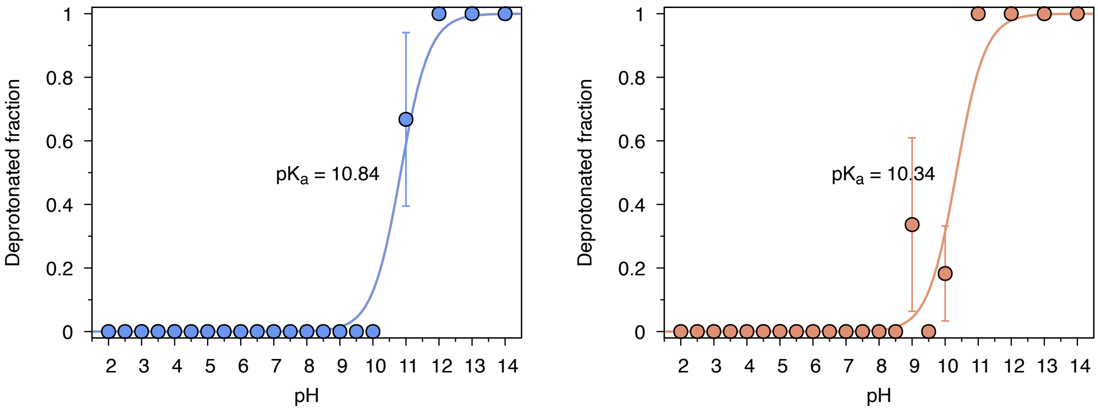

Figure S13: Deprotonated fraction of E18 for chain A (blue, left) and chain B (salmon, right) in function of the pH value. We observe a strong pK_a shift: 10.84 [10.52, 11.50] for chain A and 10.34 [10.02, 10.53] for chain B, in qualitative agreement with previous experimental[2] (which considers E18 protonated over the C106 protonation range) and computational[3] works (where E18 pK_a has been estimated $>$ 14 for deprotonated CYS106).

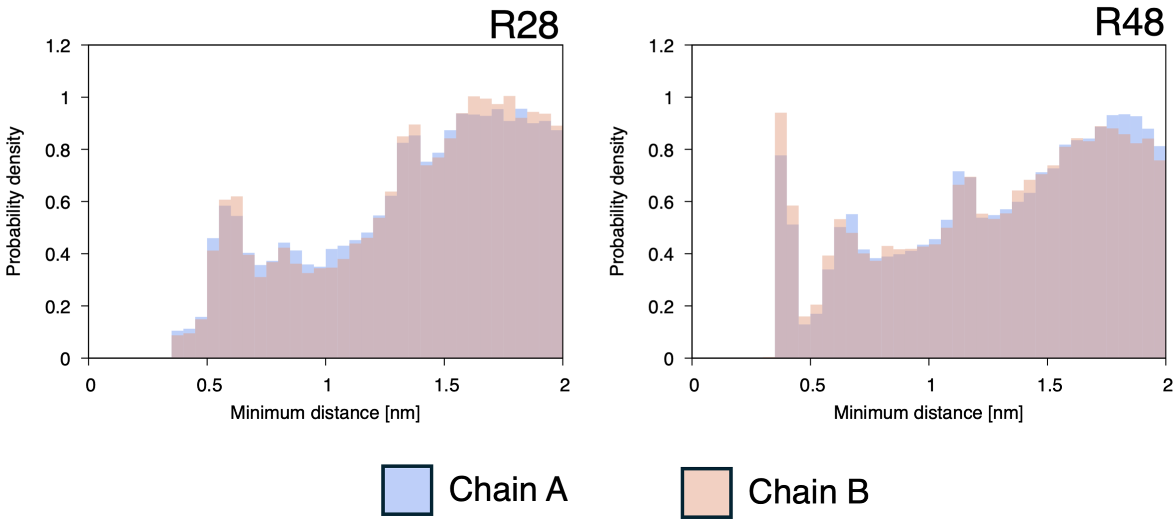

Figure S14: Distribution of the minimum distance between chloride ions and nearby arginine side chains in DJ-1. Minimum Cl^{-}–C_{ζ} distances were computed for Arg28 and Arg48 in chains A and B and pooled over the transition-region simulations from pH 3.5 to 6.0. The overlapping distributions for the two protomers indicate no evident chain-specific enrichment of chloride ions near these positively charged residues, suggesting that asymmetric anion binding is unlikely to explain the different apparent Cys106 pK_a values.

References

(1) Wilson, C. J.; Gapsys, V.; de Groot, B. L. Improving pK_a Predictions with Reparameterized Force Fields and Free Energy Calculations. *Journal of Chemical Theory and Computation* **2025**, *21*, 4095–4106.

(2) Witt, A. C.; Lakshminarasimhan, M.; Remington, B. C.; Hasim, S.; Pozharski, E.; Wilson, M. A. Cysteine pKa Depression by a Protonated Glutamic Acid in Human DJ-1. *Biochemistry* **2008**, *47*, 7430–7440.

(3) Wilson, C. J.; de Groot, B. L.; Gapsys, V. Resolving coupled pH titrations using alchemical free energy calculations. *Journal of Computational Chemistry* **2024**, *45*, 1444–1455.
